# Incidental ileal schwannoma mimicking acute appendicitis: an intraoperative diagnostic challenge

**DOI:** 10.1093/jscr/rjag311

**Published:** 2026-04-28

**Authors:** Hafiz Syed Zaigham Ali Shah, Muhammad Musaab, Syed Salman Hussain Zaidi, Ayesha Mahmood, Syed Saqib Ali Shah, Noor-Ul-Ain Mujahid

**Affiliations:** General Surgery Department, Aberdeen Royal Infirmary/ University of Aberdeen, King's Street, Aberdeen AB24 3FX, United Kingdom; King Edward Medical University/ Mayo Hospital, Nila Gumbad Road, Lahore 5400, Punjab, Pakistan; King Edward Medical University/ Mayo Hospital, Nila Gumbad Road, Lahore 5400, Punjab, Pakistan; King Edward Medical University/ Mayo Hospital, Nila Gumbad Road, Lahore 5400, Punjab, Pakistan; King Edward Medical University/ Mayo Hospital, Nila Gumbad Road, Lahore 5400, Punjab, Pakistan; Aberdeen Royal Infirmary, University of Aberdeen, King's Street, Aberdeen AB24 3FX, United Kingdom; King Edward Medical University/ Mayo Hospital, Nila Gumbad Road, Lahore 5400, Punjab, Pakistan; King Edward Medical University/ Mayo Hospital, Nila Gumbad Road, Lahore 5400, Punjab, Pakistan

**Keywords:** ileal schwannoma, acute appendicitis mimic, S-100 protein, spindle cells, neurocutaneous lesions

## Abstract

Schwannomas are benign peripheral nerve tumors, with gastrointestinal involvement being rare and ileal origin exceptionally uncommon. Their nonspecific symptoms often mimic acute abdominal conditions, complicating preoperative diagnosis. A 40-year-old female presented with acute right iliac fossa pain, nausea, vomiting, and fever, initially suggestive of acute appendicitis. Ultrasonography showed a blind-ending tubular structure. During emergency appendicectomy, the appendix appeared normal, but an exophytic ileal mass was identified. Segmental ileal resection with primary anastomosis was performed. Histopathology revealed spindle cells with Antoni A and B areas, and immunohistochemistry was strongly positive for S-100, confirming schwannoma. Postoperative computed tomography of abdomen and pelvis showed no additional lesions. The patient also had multiple cutaneous lesions and was referred to dermatology for further evaluation. Ileal schwannoma is a rare cause of acute abdomen and may mimic appendicitis. Histopathology and immunohistochemistry are essential for diagnosis, while complete surgical excision is curative. Postoperative imaging ensures comprehensive assessment.

## Introduction

Schwannoma, also known as a Schwann cell tumour, is a benign neoplasm arising from Schwann cells that insulate peripheral nerve fibers [1]. Schwannomas are among the most common peripheral nerve sheath tumors, but their exact proportion of all benign tumors (including soft tissue and others) is *not well defined* [2]. Although schwannomas may occur anywhere in the body, they most frequently originate in the head and neck region, with vestibular schwannoma of the eighth cranial nerve being the most commonly reported presentation [3].

Schwannomas of the gastrointestinal tract are rare and are most often located in the stomach or colon [4]. Intestinal schwannomas may present with nonspecific symptoms and can mimic acute inflammatory conditions such as psoas abscess or acute appendicitis [5]. We report a rare case of ileal schwannoma discovered incidentally during surgery in a 40-year-old female who presented with clinical features suggestive of acute appendicitis.

## Case report

**Figure 1:**
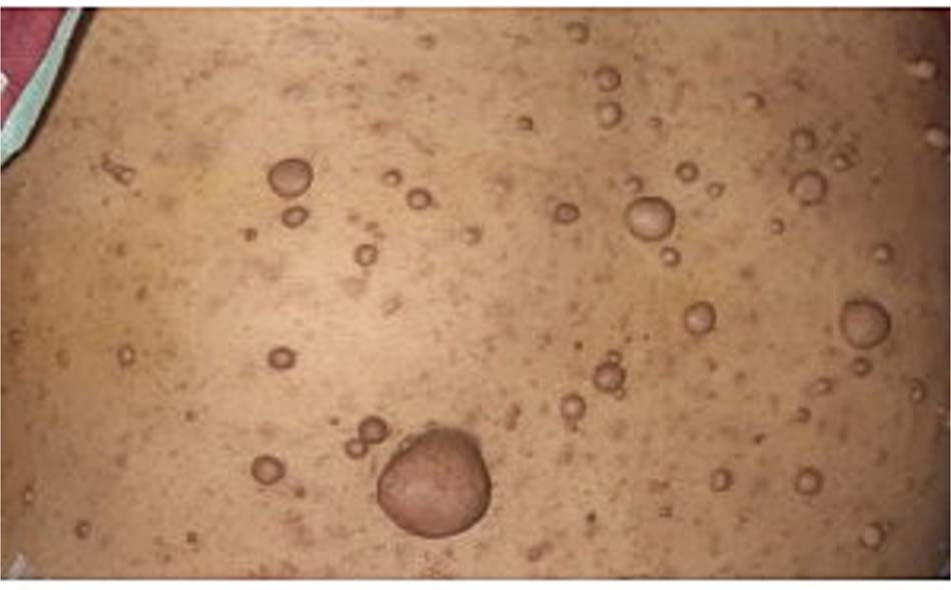
Multiple cutaneous lesions.

A 40-year-old female presented to the emergency department with severe abdominal pain that began in the periumbilical region and migrated to the right iliac fossa over 12 hours. The pain was associated with fever, nausea, and four episodes of vomiting. On physical examination, marked tenderness with rebound tenderness was noted in the right iliac fossa, raising clinical suspicion of acute appendicitis. The patient also had multiple cutaneous lesions (Fig. 1); however, no prior evaluation for an underlying neurocutaneous disorder had been performed.

**Figure 2:**
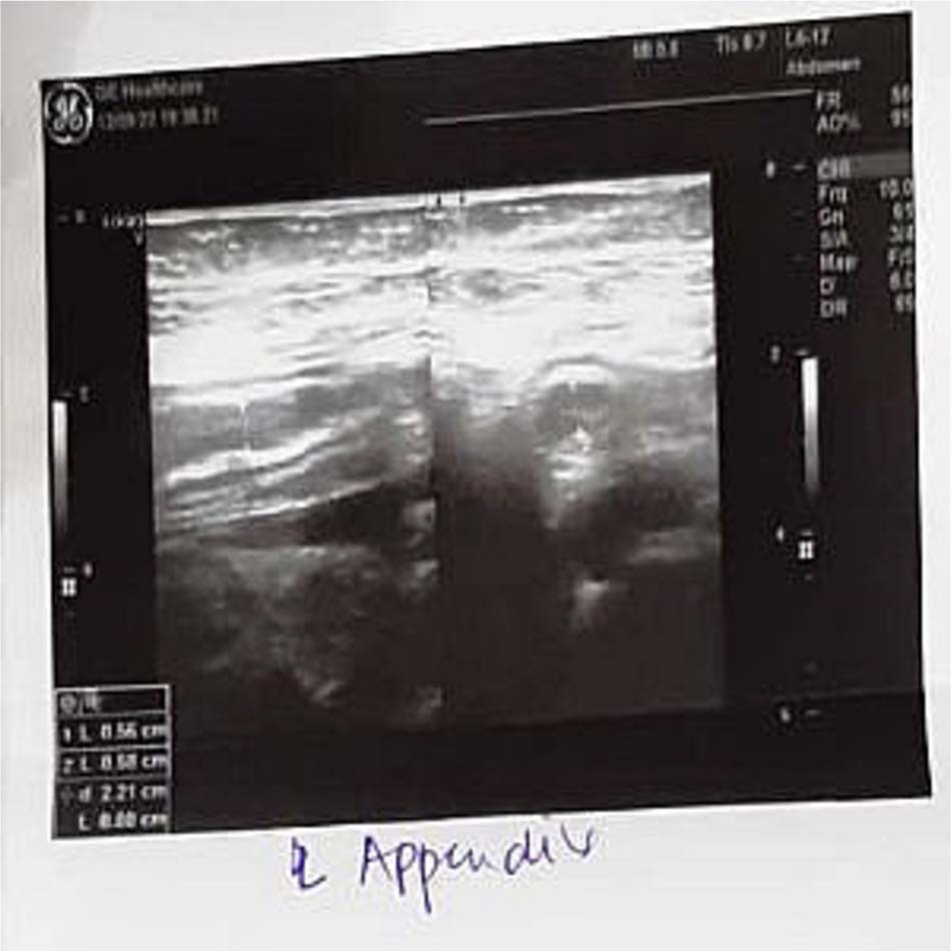
Ultrasound.

Laboratory investigations revealed leukocytosis (15 000 cells/μl). Abdominal ultrasonography demonstrated free fluid in the right iliac fossa and a 0.58-cm blind-ending tubular structure, along with enlarged mesenteric lymph nodes, the largest measuring 2.1 × 1.2 cm (Fig. 2). The uterus was anteverted, measuring 7.5 × 5 × 4 cm, and both ovaries appeared normal. The patient had no history of inflammatory bowel disease or tuberculosis. Serum beta-hCG was negative, and her menstrual cycles were regular.

Based on the clinical presentation, laboratory findings, and imaging, a provisional diagnosis of acute appendicitis was made. A contrast-enhanced computed tomography (CT) scan was not performed due to strong clinical suspicion. The patient was started on antibiotics, consented for surgery, and underwent an appendicectomy on the same day.

**Figure 3:**
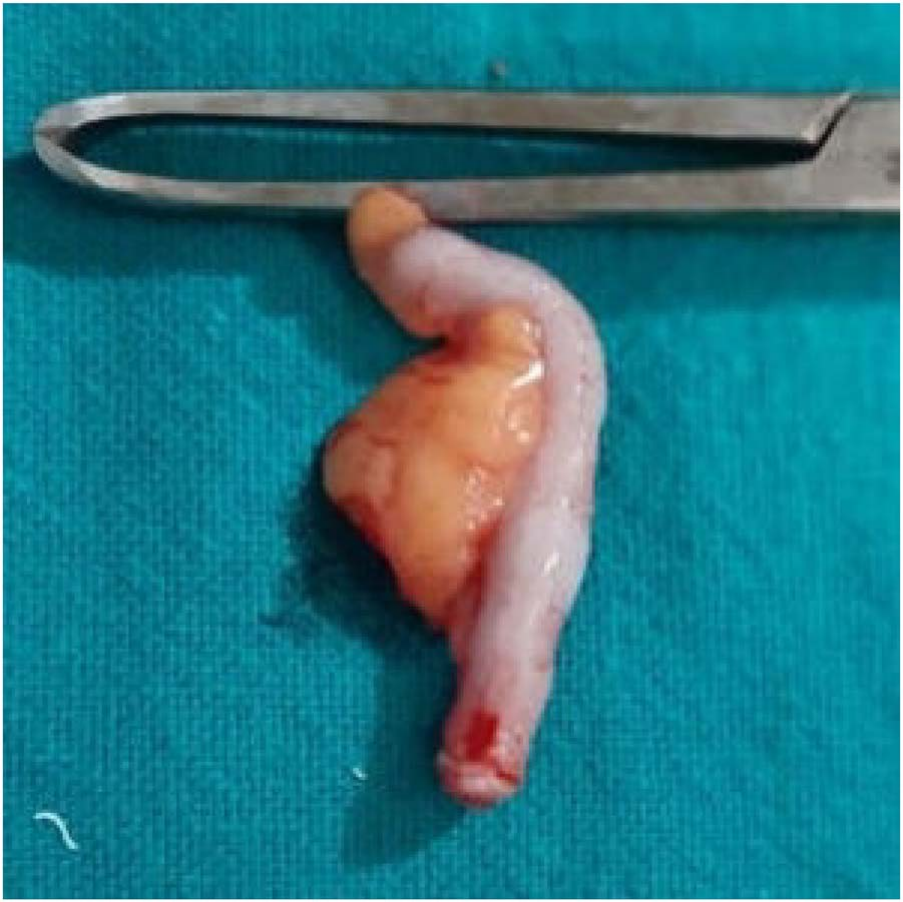
Normal appendix.

**Figure 4:**
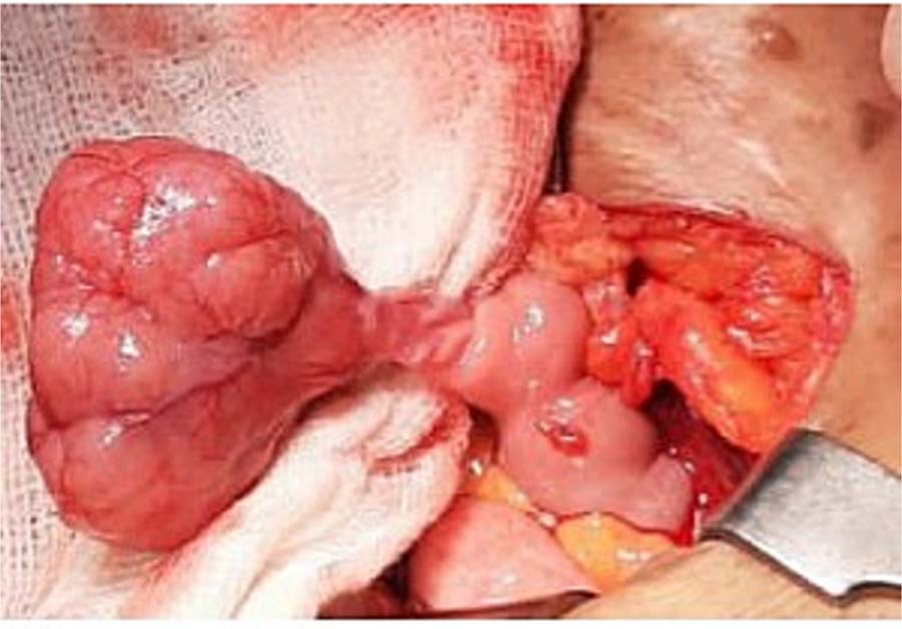
Ileal exophytic mass.

Intraoperatively, the appendix appeared grossly normal (Fig. 3). Both ovaries and fallopian tubes were normal, and no significant mesenteric lymphadenopathy was observed. However, an exophytic mass was identified on the ileum ~75 cm proximal to the ileocecal junction, along with ~100 ml of reactive peritoneal fluid (Fig. 4). A 10-cm segment of the affected ileum was resected with clear margins, and an ileo-ileal anastomosis was performed.

**Figure 5:**
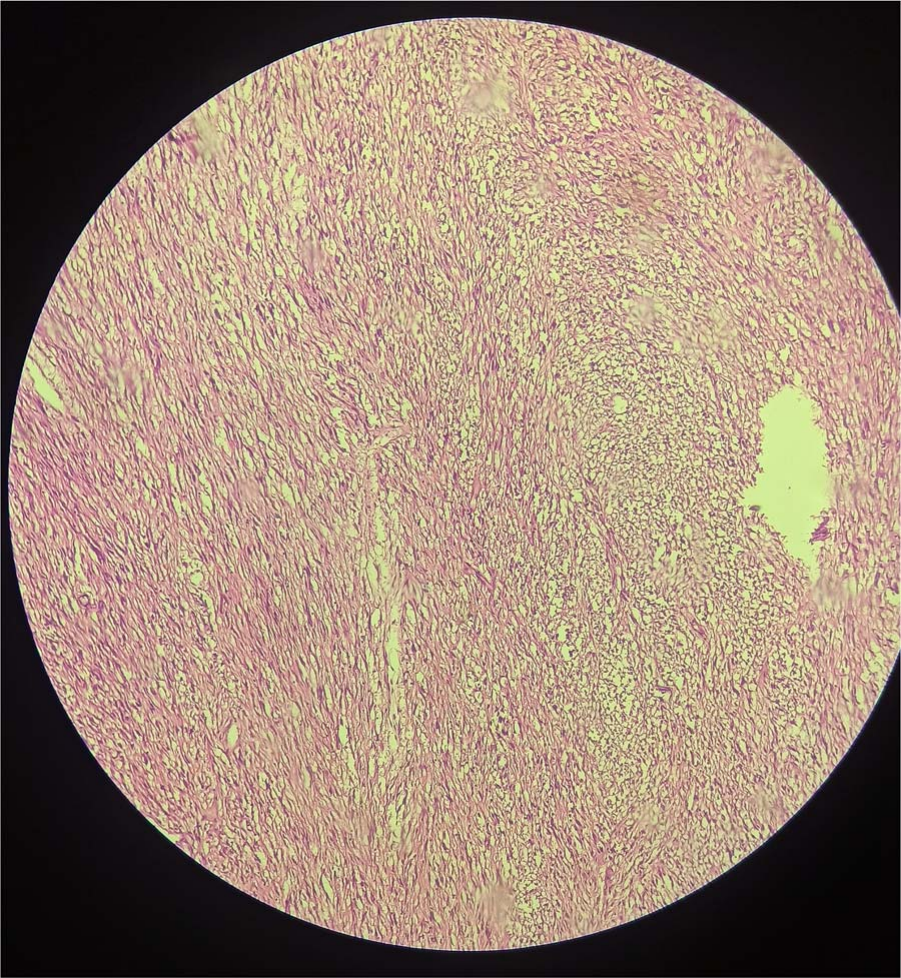
Schwannoma histology.

The postoperative course was uneventful, and the patient was discharged on the fifth postoperative day. Histopathological examination revealed an encapsulated tumor composed of elongated spindle-shaped cells arranged in a fascicular pattern, with alternating hypocellular and hypercellular areas (Fig. 5). Immunohistochemical staining was strongly positive for S-100 protein, confirming the diagnosis of schwannoma.

A contrast-enhanced CT scan of the abdomen and pelvis was performed 6 weeks postoperatively following histopathological confirmation to complete staging, which demonstrated no residual or additional lesions. Given the benign histological features, the patient was advised routine clinical follow-up with no requirement for further surveillance imaging. The patient was also referred to dermatology for further evaluation of the cutaneous lesions.

## Discussion

Gastrointestinal schwannomas constitute ~5% of mesenchymal tumours of the gastrointestinal tract [6]. These tumours typically originate from the submucosa or muscularis propria and are often covered by intact mucosa. Ileal schwannomas may present with a wide range of clinical manifestations, including abdominal pain, intussusception, gastrointestinal bleeding, and retroperitoneal abscess formation [7]. In the present case, the tumour mimicked acute appendicitis, presenting with right iliac fossa pain, tenderness, and associated systemic symptoms.

Preoperative diagnosis of ileal schwannoma is challenging due to its rarity and nonspecific presentation. Imaging modalities such as ultrasound, CT scan, magnetic resonance imaging (MRI), and angiography may aid in detection and characterization of the lesion [8]. CT and MRI are particularly useful in evaluating tumour extent and staging; however, definitive diagnosis relies on histopathological and immunohistochemical analysis of the resected specimen.

Histologically, schwannomas are characterized by spindle-shaped cells with alternating hypercellular (Antoni A) and hypocellular (Antoni B) areas. Immunohistochemical positivity for S-100 protein is a hallmark feature and confirms the diagnosis [9]. A systematic review of colorectal schwannomas reports that Ki-67 > 10% is generally considered indicative of malignant behaviour, and that mitotic rate > 5/HPF and tumour size > 5 cm are associated with higher risk of metastasis or recurrence [10].

The multiple cutaneous lesions suggested a possible neurocutaneous disorder; however, schwannomas are not typical of neurofibromatosis type 1 and are more often associated with NF2-related schwannomatosis. Gastrointestinal schwannomas are generally sporadic. The patient was referred to dermatology for further evaluation, and the significance of the skin lesions remains under investigation [11].

Complete surgical excision with clear margins followed by primary anastomosis remains the mainstay of treatment [9]. Schwannomas are generally resistant to chemotherapy and radiotherapy [12]. To date, no recurrence has been reported in cases of benign ileal schwannomas. However, lifelong surveillance is recommended in cases with malignant features [13].

## Conclusion

This case highlights the diagnostic challenge posed by ileal schwannomas presenting as acute appendicitis. Surgical exploration, followed by histopathological and immunohistochemical confirmation, is essential for diagnosis. Complete excision results in favourable prognosis, with postoperative imaging aiding comprehensive evaluation.
